# Lectures on Diseases of the Respiratory Organs, Heart and Kidneys

**Published:** 1875-06

**Authors:** 


*Lectures on Diseases of the Respiratory Organs, Heart, Lungs and Kidneys.* By Alfred L. Loomis, M. D. New York: William Wood & Co., 1875. Buffalo: H. H. Otis.

Dr. Loomis is well known to physicians and medical students through his excellent lessons in physical diagnosis, and the many admirers of that work will look for something of a high order in the one under consideration. The lecturers here presented were delivered to the class of 1874, in the Medical Department of the University of New York, and are published with but few alterations from the phonographic report of Dr. W. M. Carpenter.

The lectures are forty-four in number, and offer a concise review of the more important diseases of these parts.

We have not the time for the extended notice of the work which we should like to make, and can therefore notice but few points. Of the etiology of phthisis he gives under the head of general causes: First, Hereditary or acquired feebleness of condition; second, anti-hygienic influences; third, climate. Under that of local causes are enumerated: First, bronchitis, second, pneumonia and pleurisy, third, mechanical irritants.

Of the direct causative influence of hereditary predisposition, the lecturer has not any doubt, but does not state the case as strongly as some writers are prone to do. He takes the position that phthisis is not always transmitted from parent to offspring, but that a feebleness or vice of constitution is inherited, which renders the individual more liable to become phthisical under favoring circumstances.

Anti-hygienic influences doubtless produce a large increase in the number of phthisical patients, in fact it seems to us that they exercise a far more decided influence than climate. There can be no question but that the impure air and bad quantity and quality of food upon which many of the inhabitants of our cities live, exercises a great influence toward swelling the list.

The lectures are well arranged, and are in the main fully considered, they do not go into the disputed points of diagnostic and pathological questions for simply the reason that they are intended for the student who is not expected in his early studies to discuss these questions.

---

*The Publications of the Cincinnati Case-Record Company.*

We have received from the Cincinnati Case-Record Company, copies of their physicians Office Case-Record, pocket case-record and medical chart. The case-records are, convenient arrangements for recording the history of the patient in brief and a copy of the prescription given. The pocket edition consists of a number of prescription blanks with "stubs" for copying the prescription, on the back of which is a space systematically arranged for recording the history, beside these the record contains a visiting list, calender, table of poisons and antidotes, cash records etc. The office record is a large edition of this intended simply for office or hospital use.

The medical charts are very admirable arranged blanks for recording the pulse, temperature, respiration, and condition of the various regions so that they can be read at a glance almost. On the reverse of the chart is a space arranged for the history of the patient and a diary of the case. These aids to recording accurately the observations of practice are well conceived and are presented in an admirable shape. With their aid physicians will have no excuse for not properly recording their observations.

